# Retraction: MiR-506 Is Down-Regulated in Clear Cell Renal Cell Carcinoma and Inhibits Cell Growth and Metastasis via Targeting FLOT1

**DOI:** 10.1371/journal.pone.0345812

**Published:** 2026-03-30

**Authors:** The PLOS One Editors

After this article [1,2] was published, concerns were raised regarding Figs 3-5. Specifically,

- The following panels in Figs 3 and 5 appear to overlap with one another:
  - All four ‘ACHN’ panels of Fig 3A and 3B.
  - Both ‘48h, 786-O’ panels of Fig 3B and Fig 5D.
  - The 48h Anti-miR-Ctrl panel of Fig 3B and the Si-FLOT1 panel of Fig 5D.
  - The ‘786-O/miR-Ctrl’ panel of Fig 3C and the ‘786-O/si-NC’ panel of Fig 5E when flipped vertically.
  - The 0h 786-O panels of Fig 5D and the 0h ACHN panels of Fig 5D.
  - Both ‘Si-FLOT1’ panels of Fig 5E when one panel is flipped vertically.
- Multiple panels in Figs 3A-D of [1, 2] appear to overlap with panels in:
  - Figs 2E and 2F of [3].
  - Figs 4C-D of [4].
  - Fig 2C of [5].
  - Figs 5E and 5H of [6, retracted in 7].
- The β-actin, 786-O panel of Fig 4C appears to overlap with the GAPDH, A549 panel of Fig 5E of [8], and the GAPDH CaSki panel of Fig 7 of [11, retracted in 12], when rotated 180°.
- Multiple panels in Figs 5B and 5D-E of [1, 2] appear to overlap with panels in:
  - Figs 4A and 4C of [9].
  - Fig 2C of [5].
  - Fig 5D, when rotated 180°, and Fig 6 of [8].
  - Fig 2D of [10] when rotated 180°.

In light of the above concerns that question the reliability and integrity of the reported results and conclusions, the *PLOS One* Editors retract this article.

All authors either did not respond directly or could not be reached.

Figs 3D (panel ‘anti-miR-Ctrl/ACHN’) and 5E (panel ‘Si-FLOT1/ACHN’) in [1,2] appear similar to content previously published in [5], and Figs 5D (all ‘786-O’ and ‘0h ACHN’ panels) and 5E (panels ‘786-O/si-NC’ and ‘786-O/si-FLOT1’) in [1,2] appear similar to content previously published in [9]. Both [5] and [9] were published under a CC BY-NC license; Figs 3D (panel ‘anti-miR-Ctrl/ACHN’), 5D (all ‘786-O’ and ‘0h ACHN’ panels) and 5E (panels ‘Si-FLOT1/ACHN’, ‘786-O/si-NC’ and ‘786-O/si-FLOT1’) in [1,2] are therefore subject to the license that applies to the original articles.
